# Cross-Cultural Validation of Urdu Version KOOS in Indian Population with Primary Knee Osteoarthritis

**DOI:** 10.1155/2017/1206706

**Published:** 2017-10-25

**Authors:** Mahamed Ateef, Sivachidambaram Kulandaivelan, Mazen Alqahtani

**Affiliations:** ^1^Department of Physiotherapy, CAMS, Majmaah University, Al Majmaah 11952, Saudi Arabia; ^2^Department of Physiotherapy, GJUST, Hisar, Haryana 125001, India

## Abstract

**Purpose:**

The primary aim of this study was to translate a self-reported questionnaire (KOOS) from English to Urdu and then to see its internal consistency, agreement, test-retest reliability, and validity among primary OA knee patients.

**Methodology:**

First, KOOS questionnaire was translated from English language to Urdu through standardized cross-cultural protocol. This translated version of KOOS was administered to 111 radiographically diagnosed primary OA knee patients at two times with 48-hour interval in-between. Cronbach's alpha, floor and ceiling effect, intraclass correlation coefficient (ICC), absolute agreement %, and Spearman correlation were used to fulfill our objectives.

**Results:**

Average time to administer this questionnaire was 20 minutes. There was good internal consistency with Cronbach's alpha ranging from 0.7246 to 0.9139. The absolute agreement of each item between two tests ranged from 81.08% to 98.20%. Test-retest reliability was excellent (“*r*” ranged from 0.9673 to 0.9782). There was no ceiling effect; however less than 4% floor effect was seen in two subscales. There was significant difference that existed between different X-ray grades in all subscales meaning good content validity for disease prognosis.

**Conclusion:**

The present results show that KOOS Urdu version is a reliable and valid measure for primary OA knee patients.

## 1. Introduction

Urdu is spoken by more than 65 million people in the world who are predominantly from two Asian countries, that is, India and Pakistan. It is a national language of Pakistan and one of 22 languages recognized by Indian constitution. It is historically associated with Muslims of north Indian subcontinent [1].

OA knee is one of the most common musculoskeletal problems in middle and old aged population. The prevalence of knee OA in Indians aged 30 years or more is approximately 20% [2]. Physicians and paramedical staff use various methods/tests for diagnosis and prognosis of knee OA and to see effectiveness of interventions, that is, life-style modification that includes exercise, medicine, and surgery. The tests used in knee OA are broadly classified into subjective, that is, health related quality of life (HRQOL) questionnaires, and objective, that is, 6-minute walk test [3] categories.

Knee osteoarthritis outcome score (KOOS) is one of the disease specific questionnaires that has been widely used in literature. It has been translated into 49 languages worldwide so far. Originally developed in Swedish and for young athletes with knee injury, KOOS is a self-reported questionnaire that contains 42 questions in 05 broad domains, that is, symptoms including stiffness, pain, activities of daily living (ADL), sports-recreation, and quality of life (QOL). Each question contains 5-point Likert scale with “0” being lowest and “4” being highest. Each domain calculated by percentile with “0” means no problem and “100” means maximum problem.

English reading and understanding in India are very low which warrants the translation of self-reported questionnaires to regional languages. KOOS has been successfully translated into 8 Indian languages so far without any scientific publications on its validation in Indian population. Thus the objective of this paper is to translate the KOOS in Urdu language using cross-cultural adaptation followed by measuring internal consistency, test-retest reliability, percentage of agreement, floor–ceiling effect, content validity, and construct/criterion validity of translated version.

## 2. Methodology

The whole protocol was divided into two stages: cross-cultural adaptation and content validation. In first stage, the English version of the KOOS questionnaire was translated into Urdu version through standardized procedure recommended by Beaton et al. [4] with some modification. We translated and culturally adapted the KOOS according to Indian living standards to adapt/fit and use in Indian Urdu speaking Muslim Osteoarthritis population for the evaluation of self-rated knee functional status. In brief, stage I was done by the first author with the help of online translation portal. We skipped stages II and III recommended by Beaton et al. [4]. In stage IV, 10 Muslim experts (02 orthopaedicians, 02 rheumatologists, 02 English professors, 02 Urdu professors, and 02 religious priests) were volunteered for the review of questionnaire developed in stage I. The experts discussed and finalized that questions in sports and recreation should be supplemented by cultural activities; hence SP1 should be “squatting such as floor eating, using Indian toilet (commode)” instead of “squatting” and SP5 should be “kneeling like namas (prayer)” instead of “kneeling.” Overall, Urdu professors and religious priests simplified the language, whereas medical experts helped with the medical terminology used in the questionnaire. In stage V, questionnaire developed at the end of stage IV was administered to 20 Urdu speaking OA knee patients. All patients understood the questions and responded correctly; hence there was no modification of questionnaire at this stage.

In stage two, we evaluated the clinometric properties and validity of translated Urdu version of KOOS on primary OA knee patients. The subjects were all consecutive outpatients consulting for knee OA in two orthopaedic hospitals. The inclusion criteria were patient age of at least 40 to 75 years and primary knee OA according to the American College of Rheumatology (ACR) criteria [5], again confirmed by radiograph, and patients should be able to understand and complete the self-report questionnaires. The exclusion criteria were the presence of other significant rheumatic disease variants, low back pain, severe inflammatory arthritis as confirmed by physical examination, and intra-articular use of corticosteroids within the previous 3-month history.

A total of 119 patients (47 males and 72 females) were asked to complete the KOOS questionnaire in Urdu at outpatient department (OPD) and instructed to come two days later to fill it once again (111 patients returned [93.28% compliance rate]: 47 males and 64 females). We requested orthopaedic physicians to give minimal dose of analgesics for two days to prevent medicine effect on pain, symptoms, and ADL subscales. We decided 2-day gap as this is minimal requirement to avoid recall of answers by patients [6].

Item analysis was done using interitem correlation in which consistent values of <0.3 or >0.7 should be considered for removal of that item (question) as this indicates item irrelevance to the group (subscale) [7]. Internal consistency of individual subscales was measured using Cronbach's alpha (with less than 0.7, removal should be considered [6, 7]), two-day test-retest reliability using ICC [6, 7], percentage of agreement as absolute and >1 point variation between 1st and 2nd time [8], and floor- [lowest 0%] ceiling [highest 100%] effect of individual subscales using percentage [8]; content validity was measured by comparing degree of disease severity assessed by X-ray using one-way ANNOVA [8]; and construct/criterion validity of pain subscale was compared with VAS using Spearman (rho) correlation [6, 9]. All analysis was done in IBM-SPSS (version 21.0) software.

## 3. Results

Table 1 shows that the mean KOOS ranged from 34.15 to 51.42. There was no ceiling (100%) effect; however 2 and 4 persons have lowest (0%) score in sports and recreation and quality of life (QOL) subscales, respectively.

Table 2 shows internal consistency and test-retest reliability of Urdu KOOS questionnaire in primary knee OA patients. Cronbach's alpha (CA) ranged from 0.725 for QOL subscale (95% CI 0.634–0.798) to 0.914 for ADL subscale (95% CI 0.889–0.935). Since all subscale CA is greater than 0.7, there is no need of removal of subscale from the questionnaire. Two-day test-retest reliability of Urdu KOOS questionnaire ranged from 0.967 for symptom subscale (95% CI 0.952–0.977) to 0.986 for ADL subscale (95% CI 0.979–0.990). This table confirms that there is excellent short term test-retest reliability for KOOS questionnaire Urdu version.

Item analysis showed that 11%, 38%, 21%, 20%, and 16% interitem correlation of pain, symptoms, ADL, sports/recreation, and QOL subscales, respectively, were less than 0.3 and none of them were greater than 0.7 (see Supplement 1 in the Supplementary Material available online at https://doi.org/10.1155/2017/1206706). Subsequent analysis showed that P1 of pain subscale (involving all 4 pairs), S7 of symptom subscale (involving 4 out of 8 pairs), and ADL3 and ADL16 of ADL subscale (involving 8 and 10 out of 29 pairs) have most frequently produced interitem correlation less than 0.3 within subscale. 50% of P1, 66% of S7, 50% of ADL3, 63% of ADL16 interitem correlation were less than 0.3.

Absolute agreement between 1st and 2nd visit ranged from 88.29% to 99.10% (Supplement II). If 1 point difference was allowed, agreement increased to 98.20% to 100%. Agreement for individual items was more consistent in sports and recreation subscale and more different in symptoms and quality of life subscales. If 1 point difference was allowed, all items in sports and recreation subscale showed 100% agreement, whereas other subscales showed 98.20% to 100%.

Table 3 shows the descriptive statistics along with one-way ANNOVA for five subscales of Urdu version of KOOS in OA knee patients who were classified according to Kellgren-Lawrence (K/L) grades I–IV based on their X-ray. In order to see the content validity, we hypothesised that as the severity of OA knee increases, KOOS score will decrease which is confirmed in Table 3. Post hoc analysis showed that mean difference (MD) was −11.56 (95% CI −1.46 to −21.66, *p* = 0.014) for K/L3 compared to K/L2 and MD −13.88 (−6.58 to −21.18, *p* < 0.001) for K/L4 compared to K/L3 in KOOS-pain subscale. MD was −11.09 (−2.43 to −19.76, *p* = 0.004) for K/L4 compared to K/L3 in KOOS-symptom subscale. MD was −11.94 (−1.18 to −22.70, *p* = 0.019) for K/L3 compared to K/L2 in KOOS-ADL subscale. MD was −13.02 (−5.48 to −20.57, *p* < 0.001) for K/L4 compared to K/L3 in KOOS sports subscale. MD was −15.56 (−8.74 to −22.38, *p* < 0.001) for K/L3 compared to K/L2 and MD −18.40 (−13.48 to −23.33, *p* < 0.001) for K/L4 compared to K/L3 in KOOS-QOL subscale. There was no significant difference between subsequent other grades in all subscales, that is, between K/L1 and K/L2, K/L2 and K/L3, and K/L3 and K/L4. There was significant difference that existed between grades II and III and grades III and IV in majority of subscales meaning good content validity for disease prognosis and treatment. VAS was well correlated with KOOS-pain (*r*  −0.76) subscale meaning good construct validity.

## 4. Discussion

The present paper has reported the cross-cultural translation of Urdu version of KOOS along with its reliability and validity. We selected 11 KOOS articles in 10 different languages to compare our results with them. The languages in which KOOS has been translated are Portuguese [6], Swedish [8, 10], Dutch [9], Chinese [11], French [12], Persian [13], Japanese [14], Italian [15], Arabic [16], and Polish [17]. They used radiographically confirmed OA patients [6, 9, 12, 14] or pre/post-knee surgery patients [8–12, 17] or other knee injuries patients [13–16].

All articles reported test-retest reliability (ICC values) in their cross-cultural validation paper. The gap between test and retest varied from minimum 1 week in 6 studies [8, 11, 13–16] to 1 year [17] between the tests. The remaining 4 studies used 2–4 weeks between tests [6, 9, 10, 12]. Our test-retest reliability values (ICC 0.967–0.986) are greater than reported by literature. The possible reasons may be that the gap between the test and retest was short (only two days) and severity of OA knee was less as majority of literature used patients undergoing surgical intervention. Test-retest reliability values are affected by gap duration between the tests [14–16] and severity of the condition [9]. We choose two days between test and retest because analgesics given by physician would improve pain, function subscales of KOOS. Later responsiveness would result in improvement in KOOS subscales at 1–3 weeks rather than reliability especially grade I knee OA patients, but it would have increased the recall bias in patients.

The internal consistency of present study ranged between 0.725 and 0.914 which is similar to [6, 8, 9, 11–14] or lower than [15–17] other translated versions. We have not observed any ceiling effect, but less than 5% floor effect was observed in KOOS sports/recreation and QOL subscales. Literature reported floor effect in either sports/recreation subscale alone [6, 12, 14, 16] or in both sports/recreation and QOL subscales [8–11, 13, 15].

Our findings of agreement between the items are similar to Roos et al. [8]. Our item analysis showed P1, S7, and ADL16 as less appropriate (interitem score less than 0.3) for their respective subscale which is supported by Xie et al. [11] in Chinese version. Our findings on KOOS subscales based on radiographic grading are supported by de Groot et al. [9] who classified patients into 3 groups, that is, mild, moderate, and severe. Their [9] mild group KOOS subscales values are greater than our grade I; moderate group values are equal to our grade II and severe group values are equal to our grade III OA patients.

Strengths of this article include first Indian language paper, fairly large sample, and novel statistical treatment for validity. Limitations include weak cross-cultural translation process; KOOS results are not compared with SF-36 and did not report the responsiveness (improvement after treatment).

## 5. Conclusion

The present results show that KOOS Urdu version is a reliable and valid measure for primary OA knee Urdu speaking Indian patients.

## Supplementary Material

Supplement I: Inter-item analysis for KOOS (Urdu) subscales in Knee OA patients.
Supplement II: Agreement of responses between test (1st) and retest (2nd) administration of KOOS (Urdu) in Knee OA patients.

## Figures and Tables

**Table 1:** Descriptive values for KOOS subscales in radiographic OA knee patients (*n* = 119).

Subscale name	Mean ± SD	Range
KOOS-pain	44.68 ± 16.64	14–83
KOOS-symptom	51.42 ± 15.91	11–96
KOOS-ADL	48.61 ± 14.02	18–94
KOOS-sports/rec	35.34 ± 15.41	0–75
KOOS-QOL	34.15 ± 18.44	0–69

**Table 2:** Test-retest reliability and internal consistency of five KOOS subscales in Urdu version (*n* = 111).

Subscale	Test	Retest	ICC	Cronbach's alpha
	Mean ± SD	Mean ± SD		
Pain	44.34 ± 16.08	44.79 ± 15.92	0.9782	0.8799
Symptom	51.10 ± 15.22	51.59 ± 14.65	0.9673	0.7889
ADL	47.98 ± 12.93	47.94 ± 12.84	0.9855	0.9139
Sports/rec	35.59 ± 14.25	35.59 ± 13.80	0.9705	0.7563
QOL	34.05 ± 18.51	34.41 ± 18.28	0.9685	0.7246

**Table 3:** Urdu KOOS subscale score for four radiographic OA knee patient groups based on Kellgren-Lawrence (KL) grading.

Subscales	KL grade I (*n* = 17)	KL grade II (*n* = 15)	KL grade III (*n* = 39)	KL grade IV (*n* = 40)	*F* value (significance)
Pain	63.50 ± 7.36	55.71 ± 8.26	44.15 ± 14.20	30.28 ± 10.90	34.875^*∗∗∗*^
Symptom	63.38 ± 7.40	59.93 ± 13.81	51.77 ± 14.82	40.68 ± 13.44	12.641^*∗∗∗*^
ADL	59.69 ± 9.63	59.07 ± 10.48	47.13 ± 15.19	40.53 ± 9.69	11.279^*∗∗∗*^
Sports/rec	47.50 ± 12.11	45.00 ± 11.27	35.90 ± 11.97	22.88 ± 11.87	22.432^*∗∗∗*^
QOL	56.56 ± 6.74	48.71 ± 4.81	33.15 ± 8.32	14.75 ± 8.68	142.18^*∗∗∗*^

^*∗∗∗*^Means *p* < 0.001.
