# Supplementary material for: Cross-Cultural Validation of Urdu Version KOOS in Indian Population with Primary Knee Osteoarthritis
Source: Int J Rheumatol. 2017 Oct 25;2017:1206706. doi: 10.1155/2017/1206706 (PMC5676448; doi:10.1155/2017/1206706)
Supplement: Supplementary file 1 — Supplement I: Inter-item analysis for KOOS (Urdu) subscales in Knee OA patients. Supplement II: Agreement of responses between test (1st) and retest (2nd) administration of KOOS (Urdu) in Knee OA patients. [file 1206706.f1.pdf]

Supplement I: Inter-item analysis for KOOS (Urdu) subscales in Knee OA patients

Pain inter-item correlation [Cronbach's Alpha 0.880 (95% CI 0.845-0.910)

SNo	Item	0.1-2.0	2.1-2.89	2.9-7.0	7.1-8.0	8.1-9.0
1	P1	01	03	04		
2	P2			08		
3	P3		01	07		
4	P4			08		
5	P5			08		
6	P6			08		
7	P7	01		07		
8	P8		01	07		
9	P9		01	07		
		02	06	64		

P1-P3 0.283

P1-P8 0.217

P1-P7 0.001

P1-P8 0.242

Symptom inter-item correlation [Cronbach's Alpha 0.789 (95% CI 0.725-0.843)]

SNo	Item	0.1-2.0	2.1-2.89	2.9-7.0	7.1-8.0	8.1-9.0
1	S1	01		05		
2	S2	02	01	03		
3	S3		01	05		
4	S4		03	03		
5	S5	01	02	03		
6	S6		01	05		
7	S7	02	02	02		
		06	10	26		

S1-S7	0.131	S2-S4	0.262
S2-S5	0.180	S2-S7	0.163
S3-S5	0.276	S4-S6	0.262
S4-S7	0.233	S5-S7	0.277

ADL inter-item correlation [Cronbach's Alpha 0.914 (95% CI 0.889-0.935)]

SNo	Item	1.1-2.0	2.1-2.89	2.9-7.0	7.1-8.0	8.1-9.0
1	ADL1		01`	15		
2	ADL2	02	04	10		
3	ADL3	02	06	08		
4	ADL4	01		15		
5	ADL5		02	14		
6	ADL6	01		15		
7	ADL7			16		
8	ADL8		02	14		
9	ADL9	01	04	11		
10	ADL10		03	13		
11	ADL11		04	12		
12	ADL12	01	01	14		
13	ADL13			16		
14	ADL14	01	02	13		
15	ADL15		06	10		
16	ADL16	03	07	06		
17	ADL17		04	12		
		12	46	218		

ADL1-ADL16	0.250	ADL2-ADL9	0.136
ADL2-ADL11	0.249	ADL2-ADL12	0.221
ADL2-ADL14	0.284	ADL2-ADL15	0.268
ADL2-ADL16	0.197	ADL3-ADL9	0.210
ADL3-ADL10	0.238	ADL3-ADL11	0.226
ADL3-ADL12	0.194	ADL3-ADL14	0.163
ADL3-ADL15	0.282	ADL3-ADL16	0.230
ADL3-ADL17	0.238	ADL4-ADL16	0.179
ADL5-ADL15	0.260	ADL5-ADL16	0.283
ADL6-ADL16	0.169	ADL8-ADL9	0.267
ADL8-ADL17	0.252	ADL9-ADL16	0.247
ADL9-ADL17	0.262	ADL10-ADL15	0.266
ADL10-ADL16	0.276	ADL11-ADL16	0.237
ADL11-ADL17	0.230	ADL14-ADL15	0.286
ADL15-ADL16	0.239		

Sports inter-item correlation [Cronbach's Alpha 0.756 (95% CI 0.679-0.819)]

SNo	Item	0.1-2.0	2.1-2.89	2.9-7.0	7.1-8.0	8.1-9.0
1	SP1		01	03		
2	SP2			04		
3	SP3		01	03		
4	SP4		01	03		
5	SP5		01	03		
			04	16		

SP1-SP5 0.248

SP3-SP4 0.254

QOL inter-item correlation [Cronbach's Alpha 0.725 (95% CI 0.634-0.798)]

SNo	Item	0.1-2.0	2.1-2.89	2.9-7.0	7.1-8.0	8.1-9.0
1	QOL1	01		02		
2	QOL2	01		02		
3	QOL3			03		
4	QOL4			03		
		02		10		

QOL1-QOL2 0.194
