# Supplementary material for: Cross-Cultural Validation of Urdu Version KOOS in Indian Population with Primary Knee Osteoarthritis
Source: Int J Rheumatol. 2017 Oct 25;2017:1206706. doi: 10.1155/2017/1206706 (PMC5676448; doi:10.1155/2017/1206706)
Supplement: Supplementary file 2 [file 1206706.f2.pdf]

Supplement II: Agreement of responses between test (1st) and retest (2nd) administration of KOOS (Urdu) in Knee OA patients

	SP 1	SP 2	SP 3	SP 4	SP 5	Q 1	Q 2	Q 3	Q 4
0/0	-	-	-	-	-	-	1	-	-
1/1	15	8	9	10	9	18	15	12	16
2/2	39	46	35	30	33	27	23	26	24
3/3	43	36	52	46	48	46	43	39	42
4/4	8	14	10	17	17	14	18	21	26
0/1	1	-	-	-	-	1	1	-	-
1/2	2	2	1	1	-	2	1	2	-
2/3	2	3	2	6	3	2	4	5	2
3/4	1	2	2	1	1	1	3	6	1
0/2	-	-	-	-	-	-	-	-	-
1/3	-	-	-	-	-	-	1	-	-
2/4	-	-	-	-	-	-	-	-	-
0/3	-	-	-	-	-	-	-	-	-
1/4	-	-	-	-	-	-	-	-	-
0/4	-	-	-	-	-	-	1	-	-
AR (%)	94.59	93.69	95.50	92.79	96.40	94.59	90.09	88.29	97.30
R1E (%)	5.41	6.31	4.50	7.21	3.60	5.41	8.11	11.71	2.70
R>1E (%)	-	-	-	-	-	-	1.80	-	-

SP denotes sports category; Q denotes quality of life category; AR-absolute response; R1E-response with 1 error; R>1E-response with greater than 1 error

	P 1	P 2	P 3	P 4	P 5	P 6	P 7	P 8	P 9
0/0	3	1	3	1	4	1	1	-	1
1/1	21	24	19	24	28	18	21	25	21
2/2	39	43	44	42	32	40	46	42	39
3/3	37	32	30	32	33	41	33	32	38
4/4	5	7	7	6	11	5	8	9	10
0/1	-	-	-	-	-	-	1	-	-
1/2	3	3	1	4	2	2	1	-	1
2/3	-	1	5	2	1	3	-	2	-
3/4	1	-	1	-	-	-	-	-	-
0/2	-	-	-	-	-	-	-	-	-
1/3	-	-	1	-	-	-	-	1	1
2/4	-	-	-	-	-	-	-	-	-
0/3	-	-	-	-	-	-	-	-	-
1/4	2	-	-	-	-	1	-	-	-
0/4	-	-	-	-	-	-	-	-	-
AR (%)	94.59	96.40	92.79	94.59	97.30	94.59	98.20	97.30	98.20
R1E (%)	3.60	3.60	6.31	5.41	2.70	4.50	1.80	1.80	0.90
R>1E (%)	1.80	-	0.90	-	-	0.90	-	0.90	0.90

P denotes pain category; AR-absolute response; R1E-response with 1 error; R>1E-response with greater than 1 error

	S 1	S 2	S 3	S 4	S 5	S 6	S 7		
0/0	5	5	3	5	2	2	1		
1/1	32	32	35	32	27	35	32		
2/2	61	33	43	39	40	32	40		
3/3	11	28	17	28	24	35	32		
4/4	1	2	4	3	9	2	4		
0/1	-	1	1	-	1	-	-		
1/2	-	4	4	-	3	2	1		
2/3	-	4	3	3	3	1	-		
3/4	-	-	-	-	1	-	-		
0/2	-	-	-	1	1	-	-		
1/3	-	1	-	-	-	2	1		
2/4	-	-	-	-	-	-	-		
0/3	1	-	-	-	-	-	-		
1/4	-	1	1	-	-	-	-		
0/4	-	-	-	-	-	-	-		
AR (%)	99.10	90.09	91.89	96.40	91.89	95.50	98.20		
R1E (%)	-	8.11	7.21	2.70	7.21	2.70	0.90		
R>1E (%)	0.90	1.80	0.90	0.90	0.90	1.80	0.90		

S denotes symptoms category; AR-absolute response; R1E-response with 1 error; R>1E-response with greater than 1 error

	A 1	A 2	A 3	A 4	A 5	A 6	A 7	A 8	A 9
0/0	1	2	1		1	3	1	1	3
1/1	22	23	21	26	24	27	19	30	21
2/2	51	41	52	38	51	42	49	45	51
3/3	30	31	24	35	23	32	32	27	27
4/4	5	5	7	4	3	1	3	5	2
0/1	-	-	1	1	1	-	-	-	1
1/2	1	2	1	2	5	-	3	3	2
2/3	1	5	3	2	3	4	4	-	4
3/4	-	-	-	2	-	-	-	-	-
0/2	-	-	-	-	-	1	-	-	-
1/3	-	1	-	1	-	1	-	-	-
2/4	-	1	-	-	-	-	-	-	-
0/3	-	-	-	-	-	-	-	-	-
1/4	-	-	1	-	-	-	-	-	-
0/4	-	-	-	-	-	-	-	-	-
AR (%)	98.20	91.89	94.59	92.79	91.89	94.59	93.69	97.30	93.69
R1E (%)	1.80	6.31	4.50	6.31	8.11	3.60	6.31	2.70	6.31
R>1E (%)	-	1.80	0.90	0.90	-	1.80	-	-	-

A denotes ADL category; AR-absolute response; R1E-response with 1 error; R>1E-response with greater than 1 error

	A 10	A 11	A 12	A 13	A 14	A 15	A 16	A 17	
0/0	1	3	2	2	1	-	-	1	
1/1	31	23	26	22	28	25	26	23	
2/2	40	52	50	45	45	50	48	51	
3/3	31	30	29	38	27	28	30	32	
4/4	4	1	-	1	6	2	3	3	
0/1	2	1	-	-	-	-	-	-	
1/2	2	1	2	2	1	-	1	-	
2/3	-	-	2	1	2	3	3	1	
3/4	-	-	-	-	-	1	-	-	
0/2	-	-	-	-	1	-	-	-	
1/3	-	-	-	-	-	1	-	-	
2/4	-	-	-	-	-	1	-	-	
0/3	-	-	-	-	-	-	-	-	
1/4	-	-	-	-	-	-	-	-	
0/4	-	-	-	-	-	-	-	-	
AR (%)	96.40	98.20	96.40	97.30	96.40	94.59	96.40	99.10	
R1E (%)	3.60	1.80	3.60	2.70	2.70	3.60	3.60	0.90	
R>1E (%)	-	-	-	-	0.90	1.80	-	-	

A denotes ADL category; AR-absolute response; R1E-response with 1 error; R>1E-response with greater than 1 error
